# Supplementary material for: Deep-learning tool for early identification of non-traumatic intracranial hemorrhage etiology and application in clinical diagnostics based on computed tomography (CT) scans
Source: PeerJ. 2025 Feb 26;13:e18850. doi: 10.7717/peerj.18850 (PMC11871901; doi:10.7717/peerj.18850)
Supplement: Supplemental Information 1 [file peerj-13-18850-s001.pdf]

Supplementary Material

Table e-1. The accuracy of the different CNN models for model selection.

	An	Ht	AVM	MMD	CM	Others	Pooled Accuracy
AlexNet	0.9331 (4th)	0.9397 (2nd)	0.8298 (3rd)	0.8113 (2nd)	0.8432 (4th)	0.7142 (1st)	0.7238 (3rd)
ResNet	0.9450 (1st)	0.9333 (4th)	0.8648 (1st)	0.7985 (3rd)	0.8829 (2nd)	0.7127 (2nd)	0.7238 (3rd)
SENet	0.9360 (3rd)	0.9394 (3rd)	0.8075 (4th)	0.7888 (4th)	0.8627 (3rd)	0.6969 (4th)	0.7383 (1st)
ICHNet	0.9395 (2nd)	0.9457 (1st)	0.8606 (2nd)	0.8466 (1st)	0.8983 (1st)	0.7108 (3rd)	0.7383 (1st)

Table e-2. The running time, GPU memory usage, and number of Model Paramaters for each model.

	AlexNet	ResNet	SENet	ICHNet
Speed (50 iterations)	~11.5 seconds	~66 seconds	~67.5 seconds	~35 seconds
Runtime Memory	2087 MB	8919 MB	9037 MB	4441 MB
Parameter Size	~58M	~133M	~133M	~135M

Table e-3. Comparison of deep learning and each rater on pooled accuracy in task 1 & 2.

	Task 1		Task 2	
	Pooled Accuracy	P	Pooled Accuracy	P
Deep Learning	0.760		0.760	
Rater 1	0.705	<0.05	0.760	0.447
Rater 2	0.720	<0.05	0.765	0.277
Rater 3	0.640	<0.05	0.725	<0.05
Rater 4	0.760	<0.05	0.760	0.885
Rater 5			0.640	<0.05
Rater 6			0.700	<0.05

Table e-4. Overall accuracy performance comparison between unaugmented and augmented results of each rater.

	Rater1	Rater2	Rater3	Rater4	Rater5	Rater6
Unaugmented	0.760	0.765	0.725	0.760	0.640	0.700
Augmented	0.810	0.825	0.785	0.800	0.765	0.835
Increment	0.05	0.06	0.06	0.04	0.125	0.135

Table e-5. Distribution of CT scanner manufactures in datasets.

	Siemens	GE	Phillips
Development dataset	1512	356	0
TT200	108	42	50
SD98	6	92	0

Table e-6. Comparison between confusion matrices without and with AI augmentation of each rater.

Rater1 ----- Without Augmentation

True \ Predict	Aneurysm	Hypertensive hemorrhage	AVM	MMD	CM	Others
Aneurysm	59	2	2	3	0	4
Hypertensive hemorrhage	0	65	1	3	5	1
AVM	0	2	7	0	0	2
MMD	0	5	1	4	1	1
CM	0	1	3	0	10	0
Others	4	5	1	0	1	7

Rater1 ----- With Augmentation

True \ Predict	Aneurysm	Hypertensive hemorrhage	AVM	MMD	CM	Others
Aneurysm	64	2	2	0	0	2
Hypertensive hemorrhage	0	69	1	0	4	1
AVM	0	2	6	1	0	2
MMD	0	4	3	3	0	2
CM	0	1	2	0	9	2
Others	4	2	0	0	1	11

Rater2 ----- Without Augmentation

True \ Predict	Aneurysm	Hypertensive hemorrhage	AVM	MMD	CM	Others
Aneurysm	66	0	2	2	0	0
Hypertensive hemorrhage	0	61	10	2	2	0
AVM	0	1	8	0	1	1
MMD	0	4	3	4	0	1
CM	0	1	4	0	9	0
Others	5	1	7	0	0	5

Rater2 ----- With Augmentation

True \ Predict	Aneurysm	Hypertensive hemorrhage	AVM	MMD	CM	Others
Aneurysm	68	0	2	0	0	0
Hypertensive hemorrhage	1	71	2	0	1	0
AVM	0	2	8	1	0	0
MMD	0	6	1	4	0	1
CM	0	3	2	0	8	1
Others	6	2	2	2	0	6

Rater3 ----- Without Augmentation

True \ Predict	Aneurysm	Hypertensive hemorrhage	AVM	MMD	CM	Others
Aneurysm	65	0	0	1	2	2
Hypertensive hemorrhage	0	60	10	0	4	1
AVM	0	0	5	1	2	3
MMD	1	4	3	3	1	0
CM	0	1	0	1	8	4
Others	5	3	4	0	2	4

Rater3 ----- With Augmentation

True \ Predict	Aneurysm	Hypertensive hemorrhage	AVM	MMD	CM	Others
Aneurysm	69	0	1	0	0	0
Hypertensive hemorrhage	2	69	0	0	4	0
AVM	0	2	8	1	0	0
MMD	0	5	3	3	0	1
CM	1	2	3	0	4	4
Others	6	7	0	0	1	4

Rater4 ----- Without Augmentation

True \ Predict	Aneurysm	Hypertensive hemorrhage	AVM	MMD	CM	Others
Aneurysm	68	0	1	1	0	0
Hypertensive hemorrhage	3	60	6	1	5	0
AVM	0	0	9	1	1	0
MMD	3	2	3	0	4	0
CM	0	2	0	0	11	1
Others	5	2	5	2	0	4

Rater4 ----- With Augmentation

True \ Predict	Aneurysm	Hypertensive hemorrhage	AVM	MMD	CM	Others
Aneurysm	68	1	1	0	0	0
Hypertensive hemorrhage	1	70	2	1	1	0
AVM	0	1	9	1	0	0
MMD	1	4	1	1	5	0
CM	0	1	2	0	9	2
Others	5	2	6	2	0	3

Rater5 ----- Without Augmentation							Rater5 ----- With Augmentation					
True \ Predict	Aneurysm	Hypertensive hemorrhage	AVM	MMD	CM	Others	Aneurysm	Hypertensive hemorrhage	AVM	MMD	CM	Others
Aneurysm	48	2	2	17	1	0	68	1	1	0	0	0
Hypertensive hemorrhage	2	61	4	4	4	0	1	69	1	1	0	3
AVM	0	1	8	1	1	0	0	3	7	1	0	0
MMD	0	6	1	3	2	0	0	6	4	1	0	1
CM	0	1	4	1	8	0	1	3	4	0	2	4
Others	4	7	2	4	1	0	5	6	1	0	0	6

Rater6 ----- Without Augmentation							Rater6 ----- With Augmentation					
True \ Predict	Aneurysm	Hypertensive hemorrhage	AVM	MMD	CM	Others	Aneurysm	Hypertensive hemorrhage	AVM	MMD	CM	Others
Aneurysm	61	1	1	7	0	0	68	0	1	1	0	0
Hypertensive hemorrhage	2	52	4	9	8	0	1	71	0	2	1	0
AVM	0	1	9	0	0	1	1	1	9	0	0	0
MMD	0	3	3	5	1	0	0	5	1	5	1	0
CM	2	1	0	1	9	1	0	0	4	0	7	3
Others	4	3	4	2	1	4	5	4	2	0	0	7

Figure e-1. Schematics of (a) training and (b) test pipelines.

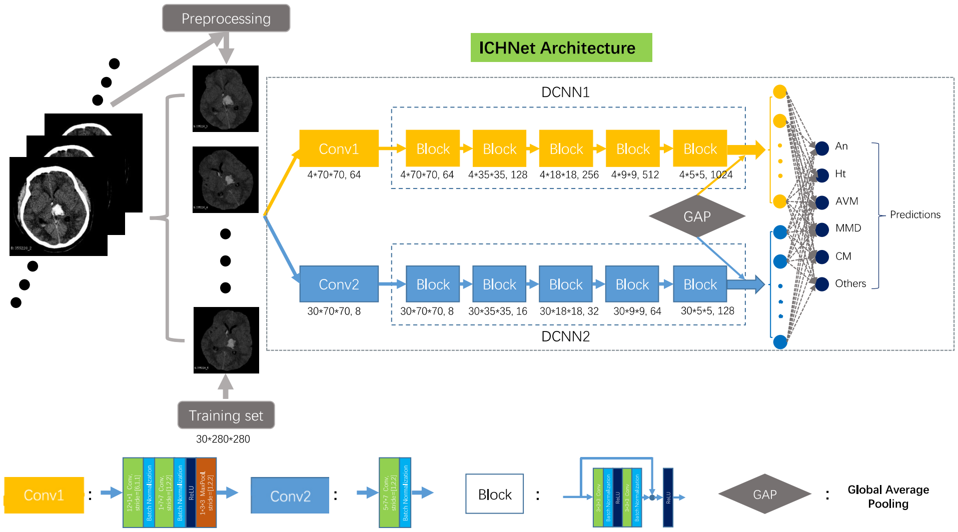

(a)

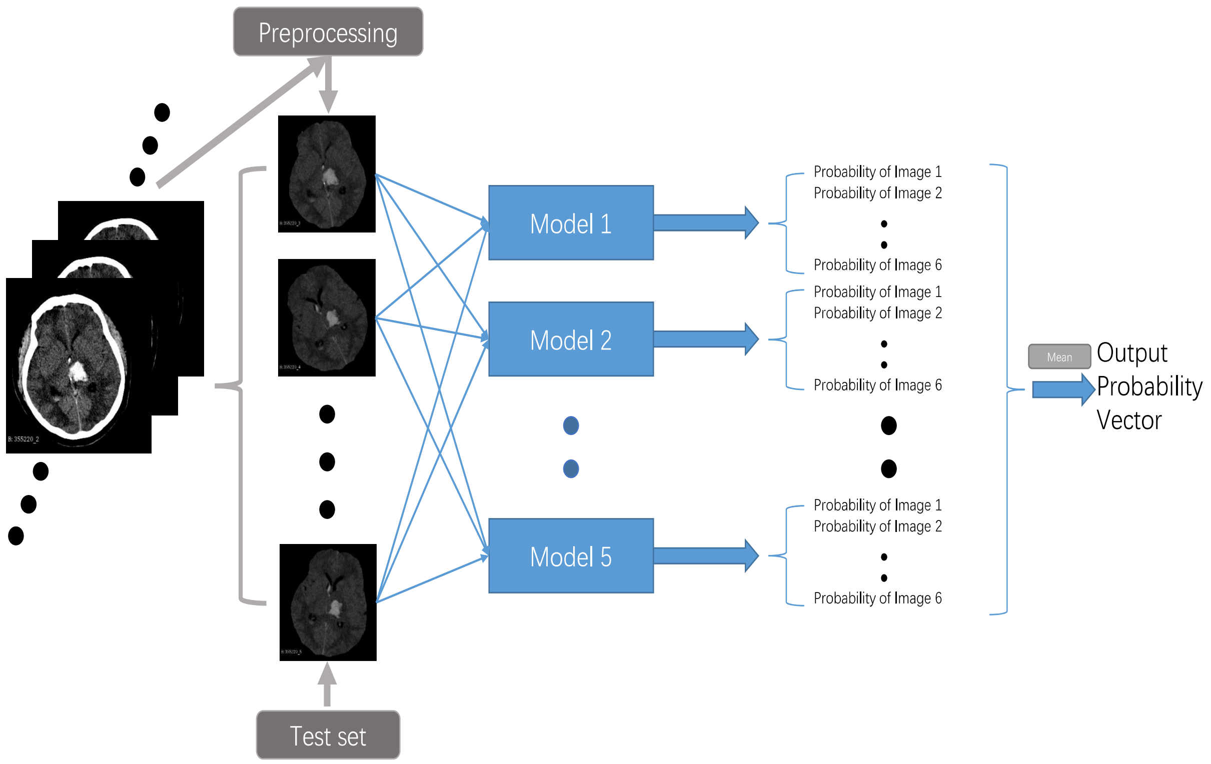
Preprocessing
Test set
Model 1
Model 2
Model 5
Probability of Image 1
Probability of Image 2
Probability of Image 6
Probability of Image 1
Probability of Image 2
Probability of Image 6
Probability of Image 1
Probability of Image 2
Probability of Image 6
Mean
Output
Probability
Vector

(b)

Figure e-2. Schematic of data collection and analysis. (I) Input DICOM images. (II) Randomly divide TT1868 dataset into five folds with four folds in the training set and one-fold in the validation set; TT200 and SD98 were prepared as test sets. (III) Preprocess the DICOM images with skull-stripping and rotation. (IV) Train the models with cross validation strategy to obtain five models. (V) Apply an ensemble strategy to the test set by averaging the outputs of each model. The final output will be the mean score.

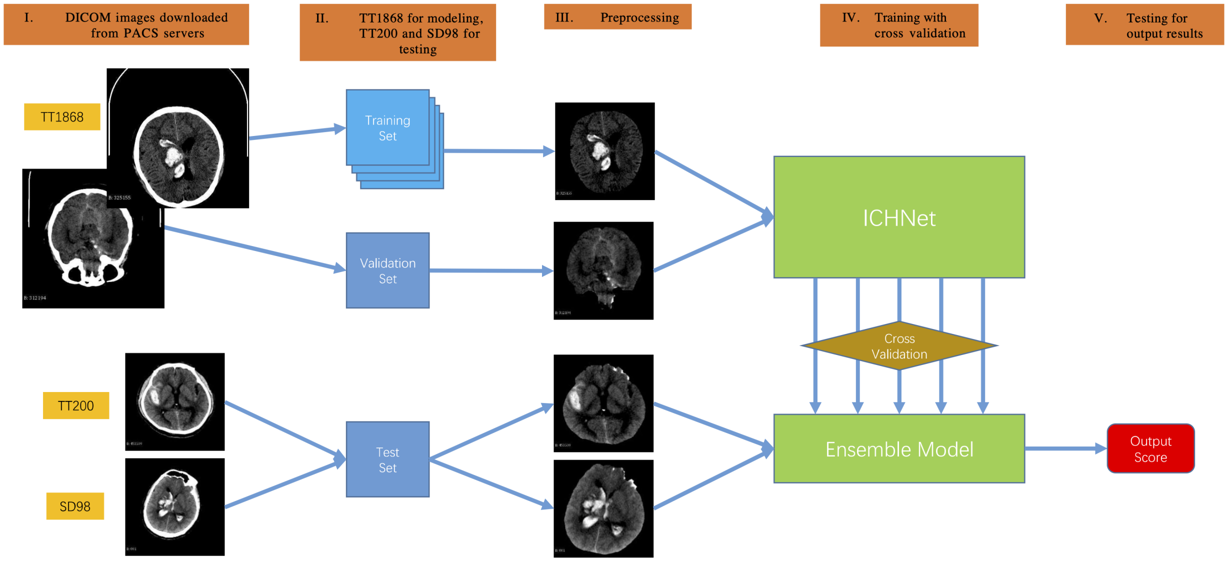

Figure e-3. ROC curves for the proposed algorithm on the TT200 dataset and the corresponding raters' results from the experiment on this prospective test data.

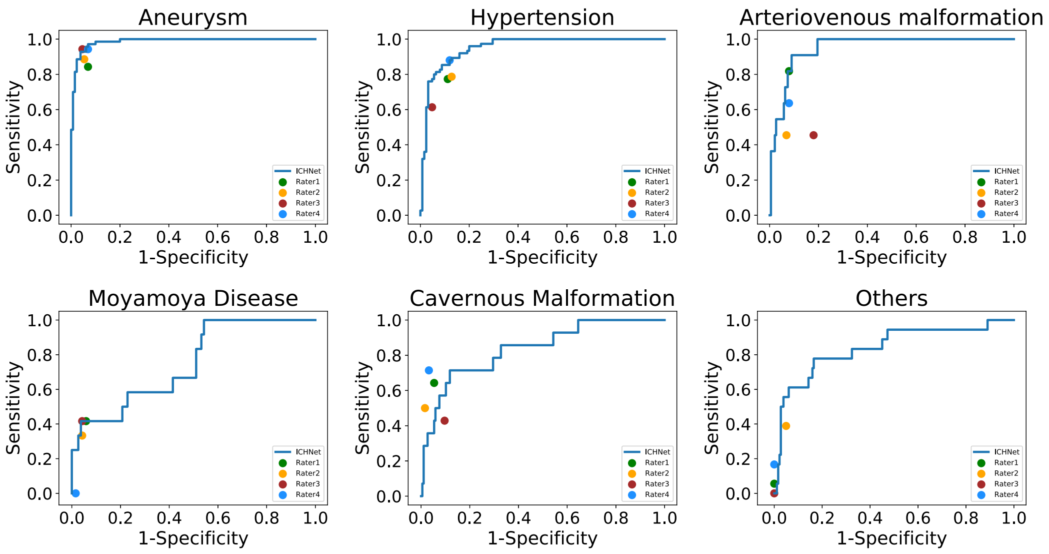

Figure e-4. ROC curves for the proposed algorithm on the TT200 dataset and the corresponding raters' results from the experiment on this prospective test data, providing the clinical history and NCCT information.

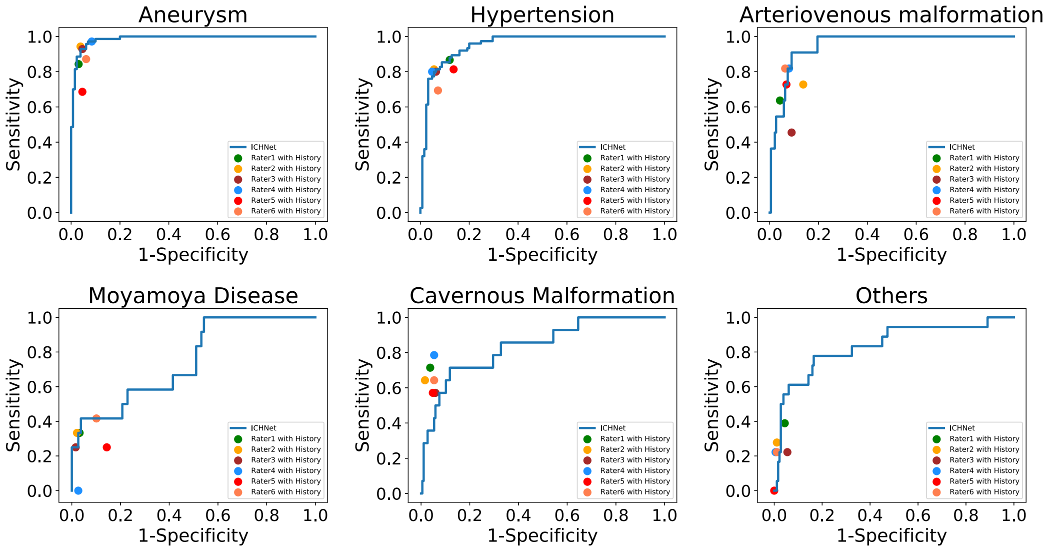

Figure e-5. Comparison of sensitivities and specificities of each raters' diagnoses without and with AI augmentation.

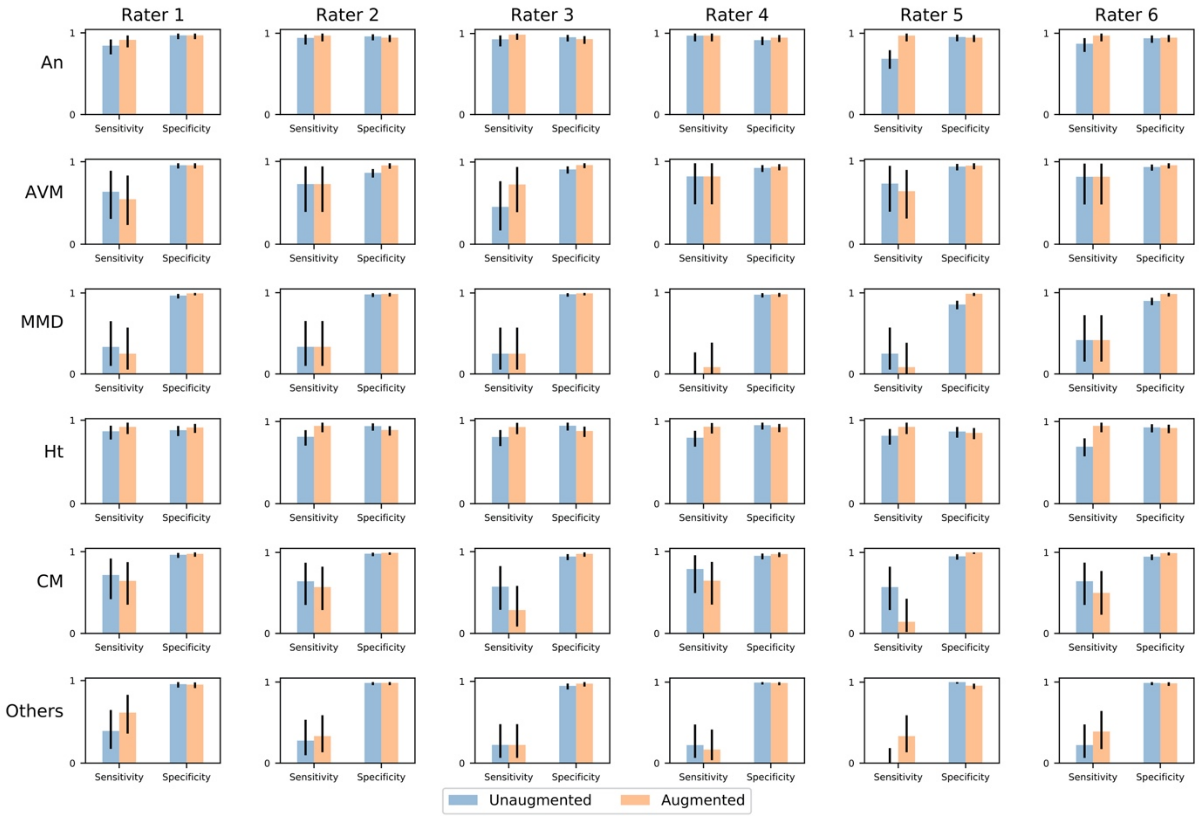

Figure e-6. Comparison between accuracy of clinicians' Task one, Task Two, and Task Three diagnoses.

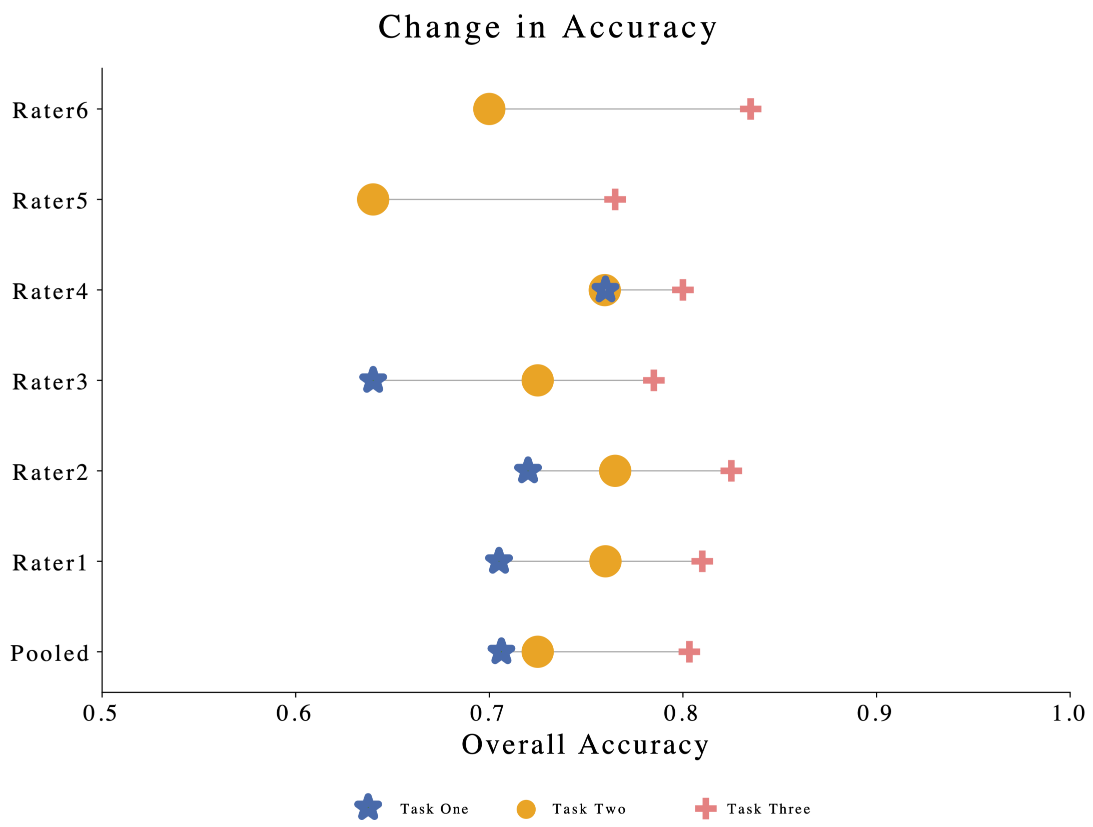

Figure e-7. (a)-(f) show spatial resolution histograms for the training and test datasets. Notably, the distributions of both the intra-slice and inter-slice resolutions of TT200 were closer to the training dataset (TT1868) than to SD98.

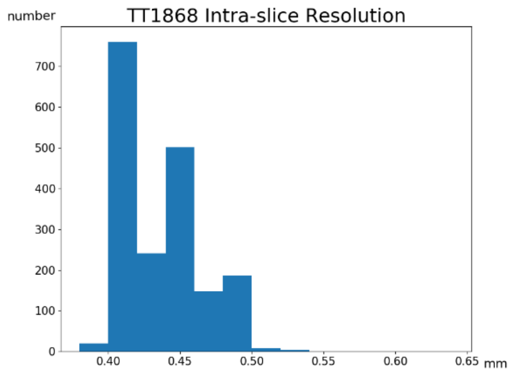

(a)

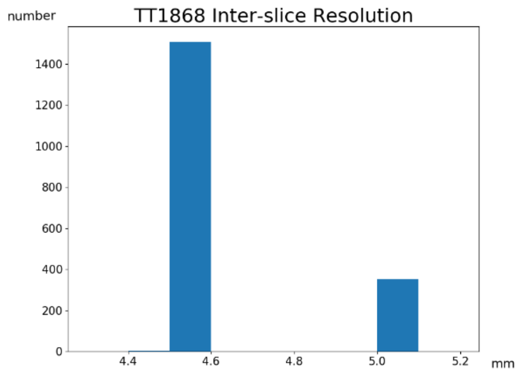

(b)

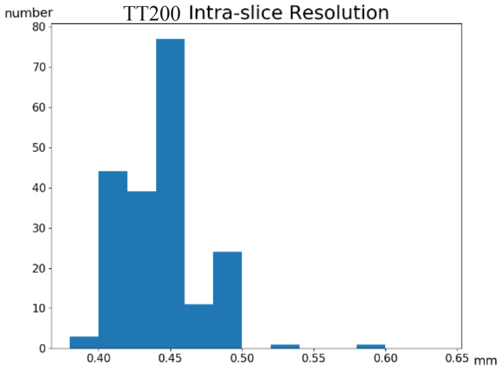

(c)

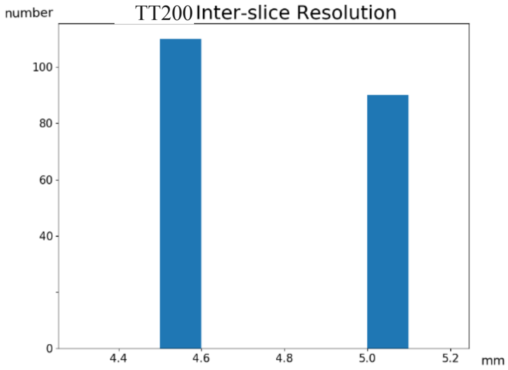

(d)

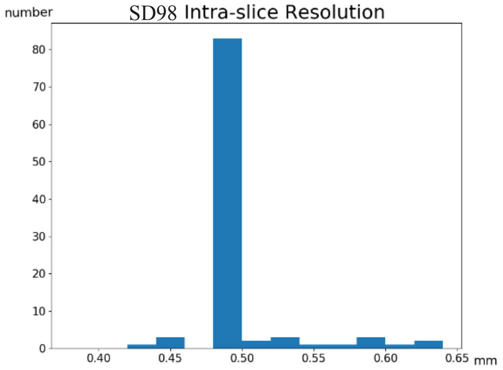

(e)

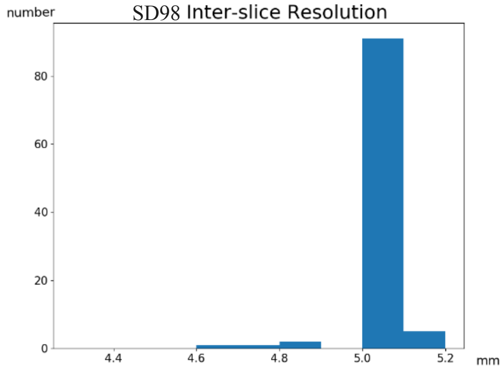

(f)

Figure e-8. (A)-(D) show images for which the proposed algorithm generated true positive predictions and enhanced the performance of raters with foreknowledge of the predictions. (E) and (F) show images for which the proposed algorithm generated erroneous predictions, but that did not degrade the raters' performance.

A

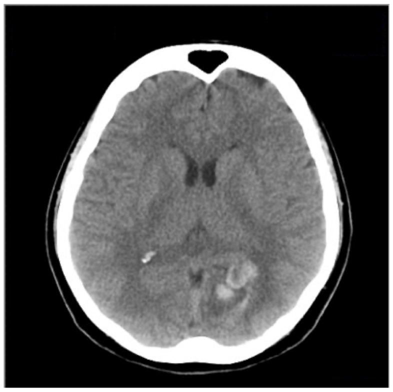

	Aneurysm	Hypertensive	AVM	MMD	CM	Others
AI	15.4%	0.8%	58.5%	24.4%	0.6%	0.3%
Ground Truth: AVM		Unaugmented: 3 AVM, 3 CM		Augmented: 6 AVM		

B

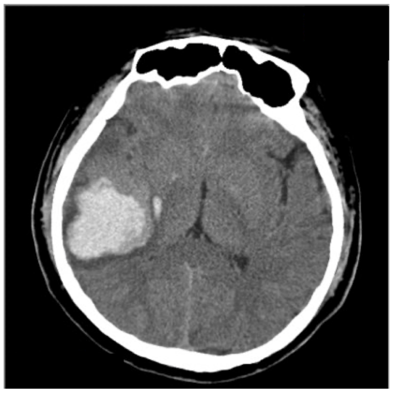

	Aneurysm	Hypertensive	AVM	MMD	CM	Others
AI	<0.1%	86.7%	7%	<0.1%	3.1%	3.2%
Ground Truth: Hypertension		Unaugmented: 6 AVM		Augmented: 3 AVM, 3 Hypertension		

C

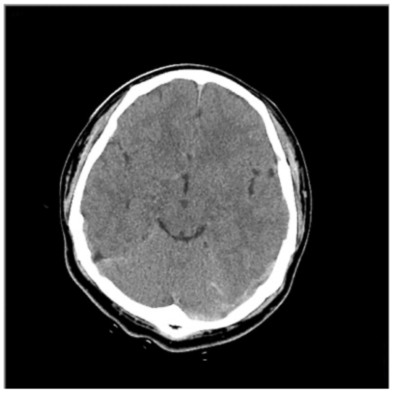

	Aneurysm	Hypertensive	AVM	MMD	CM	Others
AI	18.2%	0.2%	24.1%	<0.1%	1.1%	56.4%
Ground Truth: Others		Unaugmented: 2 AVM, 1 CM, 1 MMD, 2 Others		Augmented: 5 Others, 1 AVM		

D

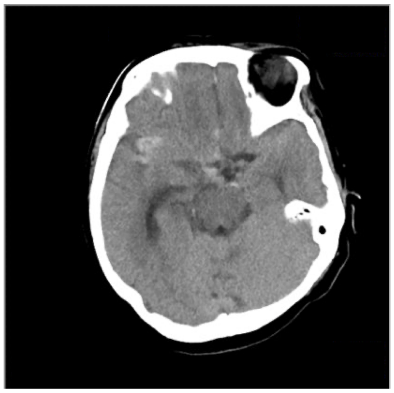

	Aneurysm	Hypertensive	AVM	MMD	CM	Others
AI	93.5%	0.1%	<0.1%	<0.1%	6.3%	<0.1%
Ground Truth: Aneurysm		Unaugmented: 3 Aneurysm, 3 MMD		Augmented: 6 Aneurysm		

E

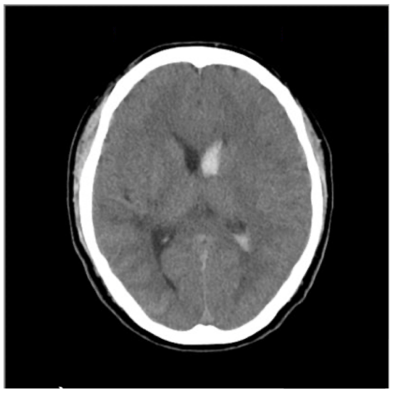

	Aneurysm	Hypertensive	AVM	MMD	CM	Others
AI	12.2%	4.9%	39.6%	19.5%	15.2%	8.7%
Ground Truth: MMD		Raters Unaugmented: 4 MMD, 1 Aneurysm, 1 Hypertension		Augmented: 4 MMD, 1 AVM, 1 CM		

F

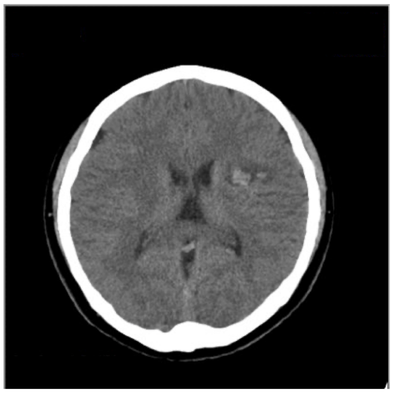

	Aneurysm	Hypertensive	AVM	MMD	CM	Others
AI	8.9%	<0.1%	44.3%	12.7%	28.4%	5.7%
Ground Truth: CM		Unaugmented: 4 CM, 2 AVM		Augmented: 5 CM, 1 AVM		
